# Trends of COVID-19 Admissions in an Italian Hub during the Pandemic Peak: Large Retrospective Study Focused on Older Subjects

**DOI:** 10.3390/jcm10051115

**Published:** 2021-03-07

**Authors:** Andrea Ticinesi, Antonio Nouvenne, Nicoletta Cerundolo, Alberto Parise, Beatrice Prati, Angela Guerra, Tiziana Meschi

**Affiliations:** 1 Geriatric-Rehabilitation Department, Azienda Ospedaliero-Universitaria di Parma, Via Antonio Gramsci 14, 43126 Parma, Italy; anouvenne@ao.pr.it (A.N.); ncerundolo@ao.pr.it (N.C.); aparise@ao.pr.it (A.P.); bprati@ao.pr.it (B.P.); angela.guerra@unipr.it (A.G.); tiziana.meschi@unipr.it (T.M.); 2 Department of Medicine and Surgery, University of Parma, 43126 Parma, Italy

**Keywords:** SARS-CoV-2, frailty, comorbidity, temporal trends, prognostic factors

## Abstract

Older multimorbid frail subjects have been severely involved in the coronavirus disease-19 (COVID-19) pandemic. The aim of this retrospective study is to compare the clinical features and outcomes of patients admitted in different phases of the outbreak in a COVID-19 hospital hub, with a particular focus on age, multimorbidity, and functional dependency. The clinical records of 1264 patients with clinical and radiological features compatible with COVID-19 pneumonia admitted in February–June, 2020, were analyzed, retrieving demographical, clinical, laboratory data, and outcomes. All variables were compared after stratification by the period of admission (first phase: rising slope of pandemic wave; second phase: plateau and falling slope), age, results of the first reverse transcriptase-polymerase chain reaction (RT-PCR) test for detection of severe acute respiratory syndrome-coronavirus-2 (SARS-CoV-2), multimorbidity (≥2 chronic diseases), and presence of disability. Factors independently associated with hospital mortality were determined by multivariate forward-selection logistic regression. Patients admitted during the second phase were older, more frequently multimorbid, disabled, and of female gender. However, on admission they exhibited milder respiratory impairment (PaO_2_/FiO_2_ 268, IQR 174–361, vs. 238, IQR 126–327 mmHg, *p* < 0.001) and lower mortality (22% vs. 27%, *p* < 0.001). Age, respiratory exchanges, positive RT-PCR test, number of chronic diseases (odds ratio (OR) 1.166, 95% confidence interval (CI) 1.036–1.313, *p* = 0.011), and disability (OR 1.927, 95% CI 1.027–3.618, *p* = 0.022) were positively associated with mortality, while admission during the second phase exhibited an inverse association (OR 0.427, 95% CI 0.260–0.700, *p* = 0.001). In conclusion, older multimorbid patients were mainly hospitalized during the second phase of the pandemic wave. The prognosis was strongly influenced by the COVID-19 phenotype and period of admission, not just by age, multimorbidity, and disability.

## 1. Introduction

The pandemic peak of coronavirus disease-19 (COVID-19) has put the Italian healthcare system into massive stress, due to high numbers of patients seeking hospital care from the end of February to June 2020 [1,2]. Hospitals were then forced to make room for medical and intensive care wards dedicated to patients with suspect or confirmed infection by Severe Acute Respiratory Syndrome coronavirus-2 (SARS-CoV-2) [3,4].

Despite the huge efforts, patients admitted with COVID-19 experienced a high burden of respiratory failure and high mortality rates. COVID-19-associated mortality is the highest in older patients [5,6], in those with multimorbidity [6,7,8,9] and cardiometabolic diseases [10,11]. Frailty and disability can represent additional risk factors for adverse outcomes in the older population [12,13,14]. 

These epidemiologic concepts, however, are not fully confirmed in clinical practice [15]. In fact, the clinical presentation and outcomes of COVID-19 are extremely variable, and do not merely depend on pre-existing risk factors even in older subjects with frailty and multimorbidity [16]. Moreover, several patients hospitalized during the pandemic peak with severe respiratory failure and chest radiology compatible with interstitial pneumonia of viral origin repeatedly tested negative at reverse-transcriptase polymerase chain reaction (RT-PCR) for SARS-CoV-2 on nasopharyngeal swabs [17,18]. These are considered false negative results of RT-PCR [19], but they could also represent a distinct phenotype of COVID-19. 

Furthermore, significant differences in clinical presentation and course of the patients hospitalized for COVID-19 across the different phases of the pandemic have been recently reported [20,21]. These observations suggest that the clinical phenotype of COVID-19 may exhibit some differences associated with the different phases of the pandemic and with the clinical and demographical profile of infected subjects. 

The primary objective of this retrospective single-center study, conducted in the COVID-19 hospital hub of an area of Northern Italy that was strongly hit by the first pandemic wave, was to compare the demographical and clinical features and outcomes of patients admitted in two different phases of the outbreak. The secondary objectives were to describe the prevalence of older age, frailty, and multimorbidity in patients admitted for suspect COVID-19, and their association with hospital mortality. 

## 2. Methods

### 2.1. Study Setting and Population

The study was conducted at the Geriatric-Rehabilitation Department of Parma University-Hospital, in the city of Parma, Emilia-Romagna region. The hospital has approximately 1200 beds, and a catchment area of 305,582 inhabitants, representing the only hospital with emergency services of the city of Parma and seventeen smaller towns in the surroundings. With the rapid surge of COVID-19 cases in Northern Italy at the end of February 2020, the Geriatric-Rehabilitation Department of the hospital was identified as the main hub for the care of COVID-19 patients needing hospitalization in ordinary wards, being separated from the main building of the hospital. The Department includes several wards with a total number of 194 beds, that can be extended up to 330 in case of need. The paths of first assessment and admission of COVID-19 patients, detailed elsewhere [4], were then built around this Department. 

Briefly, patients aged 18 or older presenting to the Emergency Department (ED) with suspect COVID-19 symptoms, such as fever, cough, dyspnea or diarrhea, or probable close contact with a positive case, were prescribed chest imaging, normally a high-resolution chest computed tomography (HRCT), in a dedicated radiology unit. Then, basing on their clinical conditions and radiology findings compatible with interstitial pneumonia, they were admitted to the Geriatric-Rehabilitation Department, irrespective of age and comorbidities, where they underwent nasopharyngeal swab for SARS-CoV-2 RT-PCR testing [4]. The assumption was that, during a pandemic wave, the pre-test probability of having COVID-19 was very high for patients with history of exposure to SARS-CoV-2, typical symptoms and chest radiology indicating interstitial pneumonia. 

HRCT images were evaluated by radiologists with experience in chest imaging, and were classified as compatible with COVID-19 pneumonia basing on the presence of ground-glass opacities alone or in combination with any of the following: areas of consolidation with nodular or mass-like aspect, organizing aspects, perilobular pattern of opacification, “reverse-halo sign” [22,23]. These criteria are coherent with those recommended by international imaging societies [24]. Other less specific abnormalities, that can be sometimes found in chest HRCT images of patients with COVID-19, including pulmonary vessel enlargement, subpleural curvilinear lines, dependent subpleural atelectasis, pleural or pericardial effusion, and mediastinal lymph node enlargement, were not considered as diagnostic criteria [25]. 

Inclusion criteria for this retrospective study were age ≥18 years old and presence of symptoms and chest HRCT findings compatible with COVID-19 interstitial pneumonia. Patients who did not undergo chest HRCT on admission and patients lacking any clinical or radiological sign of interstitial pneumonia were excluded. 

The study protocol was approved by the local Ethics Committee (ID 273/2020/OSS/AOUPR). Due to the retrospective design of the study, informed consent was obtained in written form whenever possible in compliance with the Italian law for retrospective studies.

### 2.2. Data Collection

Information on patient age, gender, medical history, symptoms and their duration, vital signs at the moment of ED arrival and on ward admission, chest imaging, laboratory tests, and clinical course was collected from each clinical record. The presence of a list of 23 specific comorbidities, including hypertension, diabetes, obesity, heart failure, chronic kidney disease (CKD), chronic obstructive pulmonary disease (COPD), asthma, previous stroke, parkinsonism, dementia, epilepsy, and cancer, was retrieved from the medical history. The total number of chronic comorbidities was also recorded, considering in this count any chronic condition, not necessarily included in the list mentioned above, with a prolonged duration or able to leave residual disability, significantly affect the quality of life, or requiring ongoing pharmacologic and non-pharmacologic treatments [26]. The CHA_2_DS_2_Vasc score was calculated as a proxy of cardiometabolic multimorbidity [27]. The number of drugs chronically taken before admission was also registered. 

The presence of partial or total dependency in performing daily activities was retrieved from the medical history, and considered as a proxy of frailty and disability, in compliance with the recommendations for evaluation of these conditions in retrospective studies [28]. 

Information collected on the findings of the chest HRCT performed on admission included the presence of ground-glass opacities, the presence of consolidations, and the COVID-19 visual score. This visual score corresponds to the percentage of lung parenchyma affected by interstitial alterations. It was calculated by radiologists with experience in chest imaging following a standardized methodology, detailed elsewhere [29]. 

The laboratory tests performed on admission included RT-PCR test for detecting SARS-CoV-2 in nasopharyngeal swab, arterial blood gas analysis, blood cell count, creatinine, urea, sodium, potassium, bilirubin, aspartate aminotransferase (AST), lactate-dehydrogenase (LDH), creatine-phosphokinase (CPK), C-reactive protein (CRP), fibrinogen and international normalized ratio (INR). Oxygen flows administered at the moment of blood analysis were also recorded, to calculate the PaO_2_/FiO_2_ ratio. 

The clinical course of each participant was then assessed taking into consideration the duration of hospital stay, the highest body temperature level, the worst oxygen saturation level and worst PaO_2_/FiO_2_ ratio, the need for non-invasive mechanical ventilation or ICU transferal. The outcome (death vs. discharge) was also registered. Finally, the drug treatments prescribed for COVID-19 management during stay (antivirals, antibiotics, hydroxychloroquine, enoxaparin, steroids) and their duration were registered for each participant. 

### 2.3. Statistical Analysis

Continuous variables were expressed as median and interquartile range (IQR). Categorical variables were expressed as percentages. The study participants were divided in two groups according to the date of admission. The first group included patients admitted during the rising slope of the pandemic wave, i.e., from 28 February to 22 March 2020. The second group included patients admitted during the plateau and falling slope, from 23 March to 10 June 2020. Mann–Whitney and chi-square tests were used for crude comparisons. Linear regression and binary logistic regression were used for age- and sex-adjusted comparisons. 

The clinical characteristics of participants were also compared by age (patients ≤70 years old vs. patients >70 years old), RT-PCR testing for SARS-CoV-2 on admission (positive vs. negative swabs), multimorbidity and functional performance. Multimorbidity was defined as the presence of two or more chronic diseases [26]. Performance was considered as a tripartite variable (i.e., autonomy in daily activities, partial autonomy, disability). Finally, a comparison between the characteristics of patients who died during hospital stay and patients who were discharged alive or transferred to other facilities was performed. The chi-square, Mann–Whitney, binary logistic regression, and linear regression tests were used for these comparisons, where appropriated. 

Stepwise multivariate logistic regression models with forward selection were then applied, to identify the factors significantly associated with hospital mortality in the entire study population (Model 1). To avoid possible selection bias, the same analysis with stepwise multivariate logistic regression (forward selection) was repeated considering patients with RT-PCR testing positive or negative for SARS-CoV-2 on admission in separate models (Models 2 and 3). 

The analyses were performed with the SPSS program, v.26 (IBM, Armonk, NY, USA), considering *p* values < 0.05 as statistically significant. 

## 3. Results

### 3.1. Temporal Trends

A total number of 1634 patients were admitted to our Department from the establishment of the COVID-19 care path (28 February 2020) to the end of the first pandemic wave (10 June 2020). Among them, 1487 clinical records were screened for inclusion. The final study population was composed of 1264 patients (711 M, 553 F) with clinical and radiological features compatible with COVID-19-related pneumonia (age range 20–99 years old).

Among these participants, 713 were admitted during the first phase of the pandemic wave (56.4%, 435 M, 278 F), while 551 (276 M, 275 F) during the second phase. The age distribution of patients admitted during the two phases is shown in Supplementary Figure S1. A comparison of the main demographical, anamnestic, clinical, and laboratory features at the moment of admission during the first and the second phase of the pandemic peak is depicted in Table 1.

Patients admitted during the second phase were older (median age 79, IQR 66–86, vs. 71, IQR 60–80 years old, *p* < 0.001), mostly of female gender (50% vs. 39%, *p* < 0.001) and disabled (27% vs. 9%, *p* < 0.001), with a higher burden of chronic diseases (comorbidities median 3, IQR 2–5, vs. 2, IQR 1–4, age- and sex-adjusted *p* < 0.001) and polypharmacy (median number of drugs 4, IQR 2–7, vs. 3, IQR 1–6, age- and sex-adjusted *p* < 0.001). The distribution of symptoms was also different, with reduced frequency of fever and cough, and higher frequency of dyspnea during the second phase. Despite similar chest HRCT visual score and duration of symptoms, patients admitted during the second phase had better PaO_2_/FiO_2_, lower frequency of positive RT-PCR testing for SARS-CoV-2 (Table 1), and different abnormalities in blood tests on ward admission (Table 2). 

Patients admitted during the second phase exhibited lower needs of oxygen support (maximum oxygen flow administered during stay 36%, IQR 28–75, vs. 50%, IQR 28–75, age-and sex-adjusted *p* < 0.001), reduced prescription of non-invasive mechanical ventilation (7% vs. 12%, *p* = 0.003) and lower hospital mortality (22% vs. 27%, *p* < 0.001) (Table 1 and Supplementary Table S1). However, the patterns of prescription of drugs against COVID-19 were also different in the second phase of the pandemic peak (Supplementary Table S1). 

### 3.2. Clinical Presentation by Age

A comparison of the anamnestic and clinical characteristics on admission between patients aged >70 and patients aged ≤70 years old is depicted in Table 3. Older patients were mostly of female gender, had a higher number of chronic comorbidities, and were mostly disabled. COVID-19 symptom distribution was also different, with lower frequency of fever and cough and higher frequency of dyspnea, resulting in lower oxygen saturation levels in room air and higher need for oxygen support (Table 3). These differences reflected also in lab tests, with lower PaO_2_/FiO_2_, hemoglobin, lymphocyte and platelet counts, and higher creatinine, sodium, D-dimer, and CRP levels in patients over 70 years old (Supplementary Table S2). 

### 3.3. Clinical Presentation by Results of RT-PCR for SARS-CoV-2 Detection on Admission

A total number of 807 patients (339 F, 468 M) tested positive at RT-PCR for SARS-CoV-2 detection on nasopharyngeal swabs performed the day of admission. Conversely, 422 patients (193 F, 229 M) tested negative for SARS-CoV-2 despite epidemiological, clinical, and radiological features suggestive of COVID-19. Nasopharyngeal swabs could not be tested on the day of admission for 35 patients. 

A comparison of the clinical and laboratory features of SARS-CoV-2 RT-PCR negative vs. positive patients is presented in Supplementary Tables S3 and S4. Namely, despite having similar HRCT findings, negative patients had lower frequency of fever and reduced need of oxygen support, with significantly higher PaO_2_/FiO_2_ (median 270, IQR 206–362, vs. 236, IQR 126–333, mmHg, age- and sex-adjusted *p* < 0.001). They also had higher blood D-dimer and lymphocyte count, and lower serum CRP values (Supplementary Table S4). 

### 3.4. Role of Multimorbidity

The number of participants with multimorbidity (≥2 chronic diseases) was 923 (73%), with a prevalence increasing from the first (68%) to the second (81%) phase of the pandemic wave. A comparison of the clinical characteristics between patients with and without multimorbidity is depicted in Table 4 and Supplementary Table S5. Patients with multimorbidity were older, mostly of female gender, and disabled. The clinical presentation of COVID-19 was not significantly different between multimorbid and non-multimorbid patients (Table 4, Supplementary Table S5), but the clinical course was more severe and mortality was higher in multimorbid patients (30% vs. 12%, *p* = 0.015 adjusted for age, sex and period of admission) (Supplementary Table S6). 

### 3.5. Role of Partial or Total Dependency in Daily Activities

Data on the functional autonomy of patients at the moment of admission were available for 1251 participants. Among them, 784 (62.6%) were classified as completely autonomous in daily activities, 257 (20.6%) as having partial autonomy and 210 (16.8%) as disabled. A comparison of the clinical characteristics at admission, clinical course, and outcome across the three groups of participants is shown in Supplementary Tables S7–S9. Disabled patients were older, mostly of female gender and multimorbid (Supplementary Table S7). The clinical presentation of COVID-19 in disabled patients was characterized by lower frequency of fever and cough, and higher frequency of dyspnea, but the PaO_2_/FiO_2_ ratio upon admission was similar to patients with partial or total autonomy (Supplementary Table S7). Mortality increased across the different groups of functional autonomy (17% for patients with complete autonomy, 34% for patients with partial autonomy, and 43% for patients with disability, *p* = 0.040 adjusted for age, sex and period of admission). 

### 3.6. Factors Associated with Adverse Outcomes 

A comparison of the clinical and laboratory features of patients on admission, after stratification for outcomes (discharged/transferred to other wards or territorial facilities vs. dead during stay), is represented in Table 5 and Table 6. Patients who died during the stay were in average older, with more comorbidities and functional dependency, wider lung parenchyma involvement on chest HRCT (visual score median 45, IQR 25–70, vs. 30, IQR 15–40, age- and sex-adjusted *p* < 0.001) and much worse respiratory exchanges at the moment of admission (PaO_2_/FiO_2_ median 124, IQR 79–242, vs. 282, IQR 200–366, mmHg, age- and sex- adjusted *p* < 0.001), despite a shorter interval between symptom onset and hospital referral (median 6, IQR 3–8, vs. 7, IQR 4–10, days, age- and sex-adjusted *p* = 0.026) (Table 5 and Table 6). 

The clinical and anamnestic factors associated with hospital mortality were tested with binary logistic regression models with forward selection, depicted in Table 7. In model 1, all participants were included. In model 2, to account for the possible selection bias, only patients with RT-PCR positivity for SARS-CoV-2 on the first nasopharyngeal swab performed during the hospital stay were considered. In model 3, we tested factors independently associated with mortality in patients with negative nasopharyngeal swabs. 

Age, the number of chronic comorbidities, and disability were independently associated with mortality, together with factors related with the COVID-19 phenotype, including PaO_2_/FiO_2_ on admission, platelet count and serum LDH. Notably, admission during the second phase of the pandemic peak was inversely associated with mortality in the total population (OR 0.427, 95% CI 0.260–0.700, *p* = 0.001) and in positive patients (OR 0.512, 95% CI 0.302–0.869, *p* = 0.013). 

## 4. Discussion

This study provides an overview of the clinical characteristics and outcomes of a large group of patients admitted for suspect COVID-19 in a large academic hospital in Northern Italy during the first pandemic wave in March–June 2020. The circumstance that the Department, where participating patients were admitted, was the main hub for COVID-19 care of a large geographical district provides a unique prospective on the epidemiological and clinical trends of presentation of COVID-19 during the different phases of the pandemic wave. 

The rapid surge of COVID-19 cases in Northern Italy forced the Italian Government to take very restrictive measures to control the outbreak, made effective on the whole national territory from 10 March 2020. [2,30] The effects of such restrictive measures generally require from ten to twenty days to translate into a significant drop of the number of new cases of disease and hospitalizations [31]. Thus, we can reasonably assume that patients admitted before 23 March 2020, had been infected by SARS-CoV-2 before the adoption of the restrictive measures, while patients admitted from that date onwards were infected during the lockdown. 

We demonstrated significant differences in the demographic, anamnestic, and clinical characteristics of patients admitted during two phases of the pandemic wave. These differences may have deep implications for organization of care, since patients admitted during the second phase were older, more frail or disabled, with a higher burden of chronic illness. This circumstance suggests that older multimorbid patients may have been infected by SARS-CoV-2 with a certain delay in comparison with healthy active adult persons. 

This hypothesis should be confirmed in other studies based on community infectious disease surveillance systems, including territories larger than a single district of a single country. However, it underlines the importance of undertaking preventive measures against SARS-CoV-2 spread specifically targeted at older, frail, multimorbid subjects as soon as COVID-19 cases begin to surge. The different characteristics of COVID-19 presentation during the second phase of the pandemic wave could define a distinct disease phenotype, with lower frequency of fever and cough, higher frequency of dyspnea, despite better values of PaO_2_/FiO_2_ on admission, and different laboratory abnormalities. This phenotype was also associated with lower mortality, in spite of the increasing age and complexity of admitted patients. 

The causes of this phenomenon are unclear. Improvement in the community care of COVID-19 may have led to a better selection of patients needing hospital referral during the second phase of the pandemic wave. The reduction of absolute numbers of novel cases could also have contributed to relieve the pressure on hospital services and improve the management of COVID-19, together with better knowledge of the disease pathophysiology by the clinicians. Overcrowding of hospitals during the first weeks of the pandemic should be also considered, because it may have conditioned an unwanted temporary decrease in the standards of care [3]. 

The mortality decline could also depend on the harvesting effect, with the individuals exhibiting the highest susceptibility to SARS-CoV-2 infection being infected earlier and with more severe phenotypes [32]. However, the specific characteristics of the population admitted during the second phase makes this hypothesis improbable. 

Viral load could also be involved in explaining the mortality decline. Several studies have shown that SARS-CoV-2 viral load in respiratory specimens is significantly higher in severe than in moderate or mild COVID-19 cases [33,34,35]. Clementi et al. detected significantly reduced viral loads in nasopharyngeal swabs of Italian newly diagnosed COVID-19 cases at the end of the first pandemic wave, in comparison with the cases diagnosed in the first weeks of the emergency [36]. Such data allow hypothesizing a role of social distancing and lockdown measures in determining lower virus inocula [36]. Interestingly, similar results were confirmed also in our municipality on an unselected series of nasopharyngeal swabs [37].

The assumption that reduced viral load could be involved in explaining the lower mortality in the second phase of the pandemic wave is also supported by the finding of a reduced rate of positivity of RT-PCR for SARS-CoV-2 on the nasopharyngeal swabs performed on admission during the second phase. The presence of negative nasopharyngeal swabs in patients with clinical and radiological features of COVID-19 and documented history of contact with patients with SARS-CoV-2 infection has been reported in several studies during the earlier phases of the pandemic [19,38]. Such findings may be explained by the sampling errors or selection bias (i.e., inclusion of patients who had interstitial pneumonia of another etiology), but they may also represent a distinct phenotype of COVID-19, caused by low SARS-CoV-2 inocula and characterized by a reduced nasal clearance of the virus. Thus, the presence of negative RT-PCR on admission does not allow to rule out the presence of COVID-19 [39]. However, our data show that in these cases mortality is reduced, in comparison with the cases who immediately exhibit positive RT-PCR tests. 

Our study also underlines the role of multimorbidity and frailty in conditioning the clinical course of COVID-19. The clinical phenotype of the disease at the moment of hospital admission, including PaO_2_/FiO_2_ and blood tests, such as platelet count and LDH, is strongly associated with prognosis, as suggested by the findings of other studies specifically conducted in older persons [13,40]. However, frailty and multimorbidity, that are frequently associated and causally inter-related in older persons [41], substantially contribute to modifying the phenotype and clinical course of COVID-19, representing additional risk factors for adverse outcomes [13,40], exactly like what happens in other acute illnesses [42].

Several studies have demonstrated that chronic comorbidities represent risk factors for COVID-19-related hospital admissions and mortality [7,8,9,10,43]. A multicenter Italian study showed that the presence of three specific comorbidities, i.e., diabetes, COPD, and chronic kidney disease, was independently associated with COVID-19-related hospital mortality [44]. However, in that study the Charlson Comorbidity Index represented a better predictor of the risk of death than any single comorbidity [44]. Our study confirmed such findings, since the number of chronic comorbidities, and not the presence of any single comorbidity or the CHA_2_DS_2_Vasc score, was independently associated with mortality. 

The presence of partial or total dependency in daily activities was also another factor independently associated with mortality. These conditions can be considered as a proxy for frailty, so that our results confirm the findings of other studies showing that the Clinical Frailty Scale can predict in-hospital mortality for COVID-19 [45,46,47], in a similar way to what happens in other common infections affecting older patients [48,49]. However, the specific weight of dependency in daily activities in conditioning adverse outcomes may be inferior to the clinical phenotype of COVID-19. This concept is also suggested by the findings of two studies specifically focused on older patients hospitalized with COVID-19 [13,50]. 

Some limitations should be considered when interpreting our results. The retrospective single-center study design is the most obvious one. Selection bias cannot be excluded with certainty, because the dynamics of hospital admission during the COVID-19 pandemic peak could have been influenced by the local organization of care. The circumstance that patients were admitted based on epidemiological, clinical, and radiological criteria, and not on the positivity of RT-PCR for SARS-CoV-2, should be regarded as a possible source of bias, although the pre-test probability of having COVID-19 was very high for these patients, in a pandemic wave context. The emergency situation with massive overflow of patients to hospital should also be considered as a limitation. Furthermore, multimorbidity and frailty were assessed with rather gross tools, since the emergency situation did not allow to perform a comprehensive geriatric assessment. Finally, ICU admission could not be analyzed as a secondary endpoint of the study, due to the low number of patients transferred in such setting. 

These limitations are however counterbalanced by some relevant points of strength, including the high sample size, the circumstance that our department was the main hub of admission for suspect COVID-19 in a large geographical district and the large amount of data collected for each patient. 

## 5. Conclusions

In our experience during the first pandemic wave of COVID-19 in Northern Italy, older patients, especially frail, multimorbid, and of female gender, were more frequently hospitalized during the second phase of the outbreak and exhibited a different phenotype of the disease. This circumstance may be useful for establishing preventive measures targeted at the older population in future waves or outbreaks. Multimorbidity and dependency in daily activities were independently associated with in-hospital mortality, but the prognosis was mainly influenced by the COVID-19 phenotype, i.e., presence of respiratory failure and its severity, and period of admission during the pandemic wave.

## Figures and Tables

**Table 1:** Comparison of the demographic, anamnestic, clinical features, and outcomes of patients hospitalized during different periods of the pandemic peak.

	First Period of Pandemic Peak (28 February–22 March 2020) (*n* = 713)	Second Period of Pandemic Peak (23 March–10 June 2020) (*n* = 551)	*p*	*p* ^a^
***Demography***				
Age, years	71 (60–80)	79 (66–86)	**<0.001**	-
Female gender	278 (39)	275 (50)	**<0.001**	-
Weight, kg	80 (69–91)	74 (63–86)	**<0.001**	0.078
***Comorbidities and functional performance***				
Chronic comorbidities, number	2 (1–4)	3 (2–5)	**<0.001**	**<0.001**
CHA_2_DS_2_Vasc score	2 (1–4)	3 (2–4)	**<0.001**	0.472
Hypertension	406 (57)	336 (61)	0.174	0.472
Diabetes	142 (20)	121 (22)	0.389	0.915
Heart disease	150 (21)	160 (29)	**0.002**	0.402
Obesity	93 (13)	61 (11)	0.297	0.944
Cancer	85 (12)	110 (20)	**<0.001**	**0.010**
COPD	64 (9)	66 (12)	0.141	0.624
Dementia	64 (9)	121 (22)	**<0.001**	**0.005**
Systemic drugs, n	3 (1–6)	4 (2–7)	**<0.001**	**0.013**
Complete autonomy in daily activities	520 (73)	275 (50)	**<0.001**	**<0.001**
Complete dependency in daily activities	64 (9)	149 (27)	**<0.001**	**<0.001**
***Clinical presentation of suspect COVID-19***				
Symptom duration, days	7 (4–10)	7 (3–10)	0.160	**0.025**
Cough	383 (54)	202 (37)	**<0.001**	**<0.001**
Dyspnea	335 (47)	325 (59)	**<0.001**	**0.025**
Fever	627 (88)	413 (75)	**<0.001**	**<0.001**
Diarrhea	43 (6)	44 (8)	0.210	0.271
Other symptoms	114 (16)	105 (19)	0.161	0.095
O_2_ saturation in room air on triage, %	90 (89–91)	91 (91–92)	0.645	**0.048**
Temperature on admission, °C	37.0 (36.0–37.7)	36.0 (36.0–37.2)	**<0.001**	**<0.001**
O_2_ flows administered on admission, %	30 (21–60)	36 (21–70)	**0.019**	0.987
CT visual score, %	30 (20–50)	30 (20–50)	0.739	0.235
Consolidations on chest CT	499 (70)	413 (75)	0.083	0.081
RT-PCR positive on admission	535 (75)	298 (54)	**<0.001**	**<0.001**
***Outcome***				
Intensive care unit	43 (6)	11 (2)	**0.020**	**0.023**
Death	195 (27)	123 (22)	**<0.001**	**<0.001**

^a^*p* adjusted for age and sex with linear or binary logistic regression. Data are shown as median and interquartile range or numbers and percentages. Crude comparisons were made with Mann-–Whitney test or chi-square test, as appropriate. *p* values <0.05 are indicated in bold.

**Table 2:** Comparison of the main laboratory findings on hospital admission of patients during different periods of the pandemic peak.

	First Period of Pandemic Peak (28 February–22 March 2020) (*n* = 713)	Second Period of Pandemic Peak (23 March–10 June 2020) (*n* = 551)	*p*	*p* ^a^
***Arterial blood gas analysis***				
pH	7.45 (7.42–7.48)	7.44 (7.41–7.47)	**0.003**	0.169
HCO_3_^-^, mmol/L	25 (23–27)	25 (23–27)	0.064	0.824
pCO_2_, mmHg	36 (32–39)	36 (33–40)	0.089	0.623
pO_2_, mmHg	70 (59–85)	83 (68–106)	**<0.001**	**<0.001**
PaO_2_/FiO_2_, mmHg	238 (126–327)	268 (174–361)	**<0.001**	**<0.001**
***Clinical chemistry and hematology***				
Hemoglobin, g/dL	13.7 (12.5–14.8)	12.9 (11.4–14.2)	**<0.001**	**<0.001**
White Blood Cells, 1 × 10^9^/L	6.41 (4.81–8.97)	7.15 (5.21–9.65)	**0.001**	**<0.001**
Lymphocytes, 1 × 10^9^/L	0.90 (0.63–1.21)	0.91 (0.61–1.30)	0.385	**0.018**
Platelets, 1 × 10^9^/L	201 (161–257)	220 (166–282)	**0.002**	**0.006**
Creatinine, mg/dL	0.9 (0.7–1.1)	0.9 (0.7–1.2)	0.889	0.272
Sodium, mEq/L	137 (135–140)	138 (135–140)	0.050	0.084
Potassium, mEq/L	4.0 (3.7–4.3)	4.0 (3.6–4.4)	0.304	0.469
Total bilirubin, mg/dL	0.7 (0.5–0.9)	0.7 (0.5–0.9)	0.090	0.916
Creatine-phosphokinase, IU/L	146 (82–363)	113 (57–233)	**<0.001**	**0.029**
Lactate-dehydrogenase, IU/L	357 (280–485)	318 (248–423)	**<0.001**	**0.004**
Aspartate aminotransferase, IU/L	47 (34–73)	39 (27–60)	**<0.001**	**0.035**
D-Dimer, ng/mL	972 (629–1650)	1075 (631–2043)	0.096	0.711
INR ratio	1.21 (1.13–1.30)	1.22 (1.13–1.36)	0.120	0.059
aPTT ratio	0.96 (0.90–1.05)	0.98 (0.90–1.07)	**0.017**	0.205
Fibrinogen, mg/dL	629 (513–754)	580 (465–730)	**0.001**	**0.017**
C-reactive protein, mg/L	101 (52–167)	93 (39–154)	**0.039**	0.172

^a^*p* adjusted for age and sex with linear regression. Data are shown as median and interquartile range. Crude comparisons were made with Mann–Whitney test, as appropriate. *p* values <0.05 are indicated in bold.

**Table 3:** Comparison of the demographic, anamnestic, clinical features, and outcomes of patients hospitalized for suspect COVID-19, categorized by age (>70 years old vs. ≤70 years old).

	Age ≤ 70 N = 492	Age > 70 N = 772	*p*	*p* ^a^
***Demography***				
Female gender	177 (36)	377 (49)	**<0.001**	
Weight, kg	84 (73–97)	72 (62–83)	**<0.001**	**0.001**
***Comorbidities and functional performance***				
Chronic comorbidities, number	2 (1–3)	3 (2–5)	**<0.001**	**<0.001**
CHA_2_DS_2_Vasc score	1 (0–2)	4 (3–5)	**<0.001**	**<0.001**
Hypertension	202 (41)	531 (69)	**<0.001**	**<0.001**
Diabetes	74 (15)	192 (25)	**<0.001**	**<0.001**
Heart disease	54 (11)	262 (34)	**<0.001**	**<0.001**
Obesity	93 (19)	54 (7)	**<0.001**	**<0.001**
Cancer	49 (10)	146 (19)	**<0.001**	**<0.001**
COPD	20 (4)	108 (14)	**<0.001**	**<0.001**
Dementia	5 (1)	177 (23)	**<0.001**	**<0.001**
Systemic drugs, n	2 (0–4)	5 (3–7)	**<0.001**	**<0.001**
Complete autonomy in daily activities	464 (95)	321 (42)	**<0.001**	**<0.001**
Complete dependency in daily activities	10 (2)	199 (26)	**<0.001**	**<0.001**
***Clinical presentation of suspect COVID-19***				
Symptom duration, days	7 (5–10)	7 (3–10)	**<0.001**	**0.001**
Cough	289 (59)	298 (39)	**<0.001**	**<0.001**
Dyspnea	225 (46)	435 (57)	**<0.001**	**<0.001**
Fever	446 (91)	588 (77)	**<0.001**	**<0.001**
Diarrhea	44 (9)	46 (6)	0.153	0.139
Other symptoms	74 (15)	145 (19)	0.062	0.081
O_2_ saturation in room air on triage, %	94 (90–97)	92 (88–95)	**<0.001**	**<0.001**
Temperature on admission, °C	36.9 (36.0–37.8)	36.5 (36.0–37.4)	**<0.001**	**<0.001**
O_2_ flows administered on admission, %	28 (21–44)	36 (21–75)	**<0.001**	**<0.001**
CT visual score, %	30 (20–45)	30 (20–50)	0.158	**0.029**
Consolidations on chest CT	334 (68)	522 (68)	0.810	0.594
RT-PCR positive on admission	310 (64)	499 (67)	0.279	0.187
***Outcome***				
Intensive care unit	49 (10)	8 (1)	**<0.001**	**<0.001**
Death	37 (8)	281 (36)	**<0.001**	**<0.001**

^a^*p* adjusted for sex with linear or binary logistic regression. Data are shown as median and interquartile range or numbers and percentages. Crude comparisons were made with Mann–Whitney test or chi-square test, as appropriate. *p* values <0.05 are indicated in bold.

**Table 4:** Comparison of the demographic, anamnestic and clinical features of patients categorized according to the presence of multimorbidity.

	Patients without Multimorbidity (0–1 Chronic Diseases) (*n* = 335)	Patients with Multimorbidity (≥2 Chronic Diseases) (*n* = 923)	*p*	*p* ^a^	*p* ^b^	β Standardized or Odds Ratio (95% Confidence Interval) for Multimorbidity
***Demography***						
Age, years	61 (50–72)	78 (69–85)	**<0.001**	-	-	-
Female gender	117 (35)	434 (47)	**<0.001**	-	-	-
Weight, kg	80 (69–89)	75 (65–89)	0.135	**0.005**	**0.003**	0.120
***Anamnestic data***						
CHA_2_DS_2_Vasc score	1 (0–2)	3 (2–4)	**<0.001**	**<0.001**	**<0.001**	0.295
Systemic drugs, n	0 (0–1)	5 (3–7)	**<0.001**	**<0.001**	**<0.001**	0.468
Complete autonomy in daily activities	302 (90)	490 (53)	**<0.001**	**<0.001**	**<0.001**	0.30 (0.19–0.48)
Complete dependency in daily activities	10 (3)	203 (22)	**<0.001**	**<0.001**	**<0.001**	3.44 (1.67–7.11)
***Clinical presentation of suspect COVID-19***						
Symptom duration, days	7 (5–10)	7 (3–10)	**<0.001**	0.448	0.357	-
Cough	194 (58)	388 (42)	**<0.001**	0.133	0.227	-
Dyspnea	147 (44)	517 (56)	**<0.001**	0.295	0.396	-
Fever	302 (90)	738 (80)	**<0.001**	0.195	0.296	-
Diarrhea	27 (8)	64 (7)	0.264	0.779	0.694	-
Other symptoms	60 (18)	157 (17)	0.804	0.515	0.456	-
O_2_ saturation in room air on triage, %	94 (90–97)	92 (88–95)	**<0.001**	0.462	0.571	-
Temperature on admission, °C	37.0 (36.0–37.8)	36.5 (36.0–37.5)	**<0.001**	**0.034**	0.086	-
O_2_ flows administered on admission, %	28 (21–44)	35 (21–75)	**<0.001**	0.281	0.290	-
CT visual score, %	30 (20–40)	30 (20–50)	0.168	0.450	0.429	-
Consolidations on chest CT	228 (68)	627 (68)	0.896	0.992	0.963	-
RT-PCR positive on admission	211 (63)	618 (67)	0.198	0.153	**0.039**	1.38 (1.02–1.88)

^a^*p* adjusted for age and sex or ^b^ age, sex and period of admission with linear or binary logistic regression, as appropriate. Data are shown as median and interquartile range or numbers and percentages. Crude comparisons were made with Mann–Whitney or chi-square tests, as appropriate. Data on multimorbidity were available for 1258 of the 1264 patients included in the study. *p* values <0.05 are indicated in bold.

**Table 5:** Comparison of the demographic, anamnestic, and clinical features of patients hospitalized for suspect COVID-19, categorized according to the outcome (discharged/transferred vs. dead during stay).

	Discharged or Transferred N = 946	Dead during Stay N = 318	*p*	*p* ^a^
***Demography***				
Age	71 (59–81)	81 (75–87)	**<0.001**	-
Female gender	415 (44)	133 (42)	0.491	-
Weight, kg	78 (67–90)	73 (61–87)	**0.031**	0.732
***Comorbidities and functional performance***				
Chronic comorbidities, number	2 (1–4)	4 (2–5)	**<0.001**	**<0.001**
CHA_2_DS_2_Vasc score	2 (1–4)	4 (3–5)	**<0.001**	**<0.001**
Hypertension	519 (55)	219 (69)	**<0.001**	0.620
Diabetes	160 (17)	102 (32)	**<0.001**	**0.001**
Heart disease	198 (21)	118 (37)	**<0.001**	**0.046**
Obesity	113 (12)	35 (11)	0.756	0.089
Cancer	132 (14)	60 (19)	0.024	0.298
COPD	76 (8)	51 (16)	**<0.001**	0.145
Dementia	104 (11)	73 (18)	**<0.001**	0.198
Systemic drugs, n	3 (1–6)	5 (3–8)	**<0.001**	**0.001**
Complete autonomy in daily activities	647 (69)	139 (44)	**<0.001**	0.423
Complete dependency in daily activities	122 (13)	91 (29)	**<0.001**	**0.008**
***Clinical presentation of suspect COVID-19***				
Symptom duration, days	7 (4–10)	6 (3–8)	**<0.001**	**0.026**
Cough	470 (50)	116 (37)	**<0.001**	0.175
Dyspnea	460 (49)	201 (64)	**<0.001**	**0.004**
Fever	779 (83)	251 (80)	0.129	0.503
Diarrhea	75 (8)	13 (4)	**0.027**	0.098
Other symptoms	169 (18)	44 (14)	0.092	0.066
O_2_ saturation in room air on triage, %	94 (90–96)	89 (80–93)	**<0.001**	**<0.001**
Temperature on admission, °C	36.6 (36.0–37.5)	36.6 (36.0–37.5)	0.757	0.248
O_2_ flows administered on admission, %	28 (21–44)	75 (32–75)	**<0.001**	**<0.001**
CT visual score, %	30 (15–40)	45 (25–70)	**<0.001**	**<0.001**
Consolidations on chest CT	641 (68)	216 (68)	0.784	0.586
RT-PCR positive on admission	566 (61)	244 (81)	**<0.001**	**<0.001**

^a^*p* adjusted for sex and age with linear or binary logistic regression. Data are shown as median and interquartile range or numbers and percentages. Crude comparisons were made with Mann–Whitney test or chi-square test, as appropriate. *p* values <0.05 are indicated in bold.

**Table 6:** Comparison of the laboratory tests performed on admission of patients hospitalized for suspect COVID-19, categorized according to the outcome (discharged/transferred vs. dead during stay).

	Discharged or Transferred N = 946	Dead during Stay N = 318	*p*	*p* ^a^
***Arterial blood gas analysis***				
pH	7.45 (7.42–7.47)	7.44 (7.40–7.48)	**0.002**	**<0.001**
HCO_3_^−^, mmol/L	25 (23–27)	24 (21–27)	**<0.001**	**<0.001**
pCO_2_, mmHg	36 (33–40)	35 (31–38)	**0.001**	0.064
pO_2_, mmHg	78 (66–95)	65 (54–83)	**<0.001**	**<0.001**
PaO_2_/FiO_2_, mmHg	282 (200–366)	124 (79–242)	**<0.001**	**<0.001**
***Clinical chemistry and hematology***				
Hemoglobin, g/dL	13.4 (12.2–14.5)	13.4 (11.5–13.4)	0.320	0.365
White Blood Cells, 1 × 10^9^/L	6.54 (4.88–8.94)	7.37 (5.27–10.77)	**<0.001**	**<0.001**
Lymphocytes, 1 × 10^9^/L	0.98 (0.70–1.33)	0.69 (0.46–1.00)	**<0.001**	**<0.001**
Platelets, 1 × 10^9^/L	215 (169–273)	192 (150–245)	**<0.001**	**0.004**
Creatinine, mg/dL	0.9 (0.7–1.1)	1.1 (0.8–1.6)	**<0.001**	**<0.001**
Sodium, mEq/L	137 (135–139)	139 (135–142)	**<0.001**	**0.001**
Potassium, mEq/L	4.0 (3.7–4.3)	4.1 (3.7–4.5)	**0.005**	**0.010**
Total bilirubin, mg/dL	0.7 (0.5–0.9)	0.7 (0.5–1.0)	**0.006**	**0.018**
Creatine-phosphokinase, IU/L	125 (64–256)	170 (88–434)	**<0.001**	0.069
Lactate-dehydrogenase, IU/L	326 (256–416)	426 (300–610)	**<0.001**	**<0.001**
Aspartate aminotransferase, IU/L	41 (29–61)	55 (35–90)	**<0.001**	**<0.001**
D-Dimer, ng/mL	923 (607–1604)	1344 (854–3718)	**<0.001**	**<0.001**
INR ratio	1.21 (1.13–1.30)	1.23 (1.13–1.39)	**0.003**	0.099
aPTT ratio	0.97 (0.90–1.05)	0.99 (0.90–1.08)	**0.022**	0.969
Fibrinogen, mg/dL	596 (490–730)	612 (480–754)	0.737	0.801
C-reactive protein, mg/L	87 (37–141)	132 (74–206)	**<0.001**	**<0.001**

^a^*p* adjusted for sex and age with linear regression. Data are shown as median and interquartile range. Crude comparisons were made with Mann–Whitney test, as appropriate. *p* values <0.05 are indicated in bold.

**Table 7:** Stepwise logistic regression model with forward selection, testing factors independently associated with hospital mortality. Model 1: all participants included in the study were considered (1264 patients). Model 2: only participants who tested positive at RT-PCR for SARS-CoV-2 on admission were considered (807 patients). Model 3: only participants who tested negative at RT-PCR for SARS-CoV-2 on admission were considered (422 patients).

	Odds Ratio	95% Confidence Interval	*p* ^a^
**Model 1**			
Age classes ^b^	2.236	1.754–2.851	**<0.001**
PaO_2_/FiO_2_ on admission, mmHg	0.994	0.992–0.997	**<0.001**
RT-PCR test positive for SARS-CoV-2 on admission	2.877	1.640–5.047	**<0.001**
Admission after 3/23/2020	0.427	0.260–0.700	**0.001**
CT visual score, %	1.019	1.007–1.032	**0.003**
Lymphocyte count ^c^, 1 × 10^9^/L	0.467	0.281–0.774	**0.003**
Platelet count ^c^, 1 × 10^9^/L	0.996	0.993–0.999	**0.007**
Chronic diseases, number	1.166	1.036–1.313	**0.011**
Creatinine, mg/dL	1.291	1.045–1.595	**0.018**
Lactate dehydrogenase, IU/L	1.002	1.001–1.003	**0.023**
White Blood Cell count, 1 × 10^9^/L	1.071	1.005–1.142	**0.035**
Total dependency in daily activities	1.927	1.027–3.618	**0.041**
**Model 2**			
Age classes ^b^	2.356	1.799–3.086	**<0.001**
PaO_2_/FiO_2_ on admission, mmHg	0.993	0.990–0.996	**<0.001**
Lactate dehydrogenase, IU/L	1.002	1.001–1.004	**0.001**
Lymphocyte count ^c^, 1 × 10^9^/L	0.481	0.286–0.809	**0.006**
Admission after 3/23/2020	0.512	0.302–0.869	**0.013**
Chronic diseases, number	1.173	1.031–1.335	**0.015**
Female sex	0.549	0.329–0.915	**0.021**
**Model 3**			
Age classes ^b^	3.039	1.655–5.580	**<0.001**
CT visual score, %	1.052	1.021–1.084	**0.001**
PaO_2_/FiO_2_ on admission, mmHg	0.993	0.988–0.997	**0.003**
Creatinine, mg/dL	2.283	1.236–4.216	**0.008**
Platelet count ^c^, 1 × 10^9^/L	0.993	0.988–0.999	**0.021**

^a^ All the other clinical, anamnestic and laboratory variables with significant differences in crude comparisons between patients who died during stay and patients discharged/transferred were considered in the logistic regression model with forward selection. ^b^ Age classes were considered as follows: ≤55 years old, 56–65 years old, 66–75 years old, 76–85 years old, >85 years old. ^c^ The odds ratios for these parameters are referred to increments of 1000 cells/mcL. *p* values <0.05 are indicated in bold.

## Data Availability

Raw data are available upon reasonable request to the corresponding author, in rigorously anonymous form.

## Supplementary Materials

The following are available online at https://www.mdpi.com/2077-0383/10/5/1115/s1. Supplementary Table S1: Comparison of the clinical course and treatments against COVID-19 of patients in two different phases of the pandemic peak. Supplementary Table S2: Comparison of the main laboratory tests of patients hospitalized for suspect COVID-19 on admission, categorized by age (>70 years old vs. ≤70 years old). Supplementary Table S3: Comparison of the demographic, anamnestic, clinical features, and outcomes of patients hospitalized for suspect COVID-19, categorized by results of the RT-PCR test for SARS-CoV-2 detection on nasopharyngeal swabs performed on admission (negative vs. positive). Supplementary Table S4: Comparison of the main laboratory tests of patients hospitalized for suspect COVID-19 performed on admission, categorized by the results of the RT-PCR test for SARS-CoV-2 detection on nasopharyngeal swabs (positive vs. negative). Supplementary Table S5: Comparison of the blood analysis on admission, after categorization of participants according to the presence of multimorbidity (≥2 chronic diseases). Supplementary Table S6: Comparison of the clinical course, treatments administered, and outcomes, after categorization of the participants according to the presence of multimorbidity (≥2 chronic diseases). Supplementary Table S7: Comparison of the demographic, anamnestic, and clinical features of patients, categorized according to the class of functional autonomy. Supplementary Table S8: Comparison of the blood analysis on admission, after categorization of participants according to functional autonomy. Supplementary Table S9: Comparison of the clinical course, treatments administered, and outcomes, after categorization of participants according to functional autonomy. Supplementary Figure S1: Age distribution of patients admitted during the first (28 February–23 March 2020) and the second phase (24 March–10 June 2020) of the first pandemic wave in our institution.
