# Supplementary material for: Trends of COVID-19 Admissions in an Italian Hub during the Pandemic Peak: Large Retrospective Study Focused on Older Subjects
Source: J Clin Med. 2021 Mar 7;10(5):1115. doi: 10.3390/jcm10051115 (PMC7962097; doi:10.3390/jcm10051115)

SUPPLEMENTARY TABLE 1

Comparison of the clinical course and treatments against COVID-19 of patients in two different phases of the pandemic peak.

	**First period of pandemic peak (2/28-3/22/2020) (n=713)**	**Second period of pandemic peak (3/23-6/10/2020) (n=551)**	**P**	**P[a]**
Clinical course				
Worst O_2 saturation during stay, %	91 (85-93)	92 (90-95)	**<0.001**	**<0.001**
Maximum O_2 flows during stay, %	50 (28-75)	36 (28-75)	**0.001**	**<0.001**
Worst arterial O_2 pressure, mmHg	57 (46-67)	64 (55-74)	**<0.001**	**<0.001**
Temperature peak during stay, °C	38.0 (37.2-38.8)	37.5 (36.0-38.2)	**<0.001**	**<0.001**
Non-invasive ventilation during stay	85 (12)	39 (7)	**0.003**	0.042
Therapies against COVID-19				
Antiviral drugs	371 (52)	237 (43)	**0.002**	**0.033**
Antibiotics	692 (97)	490 (89)	**<0.001**	**<0.001**
Azithromycin	606 (85)	358 (65)	**<0.001**	**<0.001**
Hydroxychloroquine	385 (54)	408 (74)	**<0.001**	**<0.001**
Steroids	93 (13)	138 (25)	**<0.001**	**<0.001**
Enoxaparin	649 (91)	496 (90)	0.379	0.349
Fondaparinux	36 (5)	61 (11)	**<0.001**	**<0.001**

[a]P adjusted for age and sex with linear or binary logistic regression.
Data are shown as median and interquartile range or percentages. Crude comparisons were made with Mann-Whitney test or chi-square test, as appropriate.

SUPPLEMENTARY TABLE 2

Comparison of the main laboratory tests of patients hospitalized for suspect COVID-19 on admission, categorized by age (>70 years old vs ≤70 years old).

	**AGE ≤70 N=492**	**AGE >70 N=772**	**P**	**P[a]**
Arterial blood gas analysis				
pH	7.45 (7.43-7.48)	7.44 (7.41-7.47)	**<0.001**	**<0.001**
HCO_3^-, mmol/L	26 (23-27)	25 (22-27)	**<0.001**	**<0.001**
pCO_2, mmHg	36 (33-40)	36 (32-39)	0.295	0.776
pO_2, mmHg	75 (65-92)	74 (62-93)	0.603	0.747
pO_2/FiO_2	284 (185-371)	237 (124-319)	**<0.001**	**<0.001**
Clinical chemistry and haematology				
Haemoglobin, g/dl	13.7 (12.5-14.8)	13.2 (11.7-14.4)	**<0.001**	**<0.001**
White Blood Cells, $1x10^9$/L	6.45 (4.77-8.94)	6.96 (5.17-9.52)	**0.020**	**0.016**
Lymphocytes, $1x10^9$/L	0.97 (0.73-1.36)	0.86 (0.57-1.21)	**<0.001**	**<0.001**
Platelets, $1x10^9$/L	218 (173-269)	203 (159-268)	**0.011**	**0.015**
Creatinine, mg/dl	0.8 (0.7-1.0)	1.0 (0.8-1.4)	**<0.001**	**<0.001**
Sodium, mEq/L	137 (135-139)	138 (135-141)	**<0.001**	**<0.001**
Potassium, mEq/L	4.0 (3.7-4.2)	4.0 (3.7-4.4)	**0.005**	**<0.001**
Total bilirubin, mg/dl	0.7 (0.5-0.8)	0.7 (0.5-0.9)	**0.015**	**0.028**
Creatine-phosphokinase, IU/L	127 (69-270)	136 (70-303)	0.492	0.060
Lactate-dehydrogenase, IU/L	337 (264-450)	346 (264-470)	0.358	0.052
Aspartate aminotransferase, IU/L	43 (32-68)	44 (30-68)	0.674	0.051
D-Dimer, ng/ml	742 (495-1303)	1188 (761-2292)	**<0.001**	**<0.001**
INR ratio	1.21 (1.14-1.29)	1.21 (1.12-1.38)	0.055	**<0.001**
aPTT ratio	0.97 (0.91-1.04)	0.97 (0.89-1.07)	0.494	0.183
Fibrinogen, mg/dl	612 (502-754)	596 (477-730)	**0.005**	**0.014**
C-reactive protein, mg/L	94 (37-153)	100 (52-166)	0.057	**0.008**

[a]P adjusted for sex with linear regression.
Data are shown as median and interquartile range. Crude comparisons were made with Mann-Whitney test, as appropriate.

SUPPLEMENTARY TABLE 3

Comparison of the demographic, anamnestic, clinical features and outcomes of patients hospitalized for suspect COVID-19, categorized by results of the RT-PCR test for SARS-CoV-2 detection on nasopharyngeal swabs performed on admission (negative vs positive).

	RT-PCR negative on admission N=422	RT-PCR positive on admission N=807	P	P[a]
Demography				
Age	73 (61-83)	74 (62-82)	0.842	-
Female gender	193 (46)	339 (42)	0.180	-
Weight, kg	75 (65-87)	78 (65-90)	0.246	0.203
Comorbidities and functional performance				
Chronic comorbidities, number	2 (1-4)	3 (1-4)	0.190	0.287
CHA_2DS_2Vasc score	3 (1-4)	3 (1-4)	0.175	**0.030**[b]
Hypertension	240 (57)	484 (60)	0.326	0.324
Diabetes	84 (20)	169 (21)	0.896	0.962
Heart disease	93 (22)	202 (25)	0.179	0.168
Obesity	46 (11)	97 (12)	0.653	0.632
Cancer	76 (18)	121 (15)	0.131	0.153
COPD	38 (9)	89 (11)	0.174	0.225
Dementia	63 (15)	105 (13)	0.417	0.940
Systemic drugs, n	4 (1-6)	3 (1-6)	0.850	0.935
Complete autonomy in daily activities	258 (62)	513 (64)	0.344	0.485
Complete dependency in daily activities	79 (19)	120 (15)	0.140	0.389
Clinical presentation of suspect COVID-19				
Symptom duration, days	7 (3-10)	7 (4-10)	0.123	**0.005**[c]
Cough	179 (43)	385 (48)	0.061	0.060
Dyspnea	229 (55)	410 (51)	0.172	0.155
Fever	304 (73)	707 (88)	**<0.001**	**<0.001**
Diarrhoea	37 (9)	48 (6)	0.092	0.097
Other symptoms	83 (20)	128 (16)	**0.043**	**0.046**
O_2 saturation in room air on triage, %	93 (89-96)	93 (88-95)	**0.017**	**0.013**
Temperature on admission, °C	36.0 (36.0-37.2)	36.9 (36.0-37.7)	**<0.001**	**<0.001**
O_2 flows administered on admission, %	30 (21-44)	33 (21-70)	**0.026**	**0.003**
CT visual score, %	30 (20-45)	30 (20-50)	0.331	0.086
Consolidations on chest CT	283 (67)	549 (68)	0.698	0.640
Outcome				
Intensive care unit	13 (3)	40 (5)	0.291	0.257
Death	58 (14)	243 (30)	**<0.001**	**<0.001**

[a]P adjusted for sex and age with linear or binary logistic regression.
[b]CHA_2DS_2Vasc Score is higher in patients with positive swab.
[c]Symptom duration is longer in patients with negative swab.
Data are shown as median and interquartile range or numbers and percentages. Crude comparisons were made with Mann-Whitney test or chi-square test, as appropriate. Data on RT-PCR results for SARS-CoV-2 detection were available in 1229 of the 1264 patients included in the study.

SUPPLEMENTARY TABLE 4

Comparison of the main laboratory tests of patients hospitalized for suspect COVID-19 performed on admission, categorized by results of the RT-PCR test for SARS-CoV-2 detection on nasopharyngeal swabs (positive vs negative).

	**RT-PCR negative on admission N=422**	**RT-PCR positive on admission N=807**	**P**	**P[a]**
Arterial blood gas analysis				
pH	7.44 (7.41-7.47)	7.45 (7.42-7.48)	**0.027**	0.065
HCO_3^-, mmol/L	25 (23-27)	25 (23-27)	0.621	0.570
pCO_2, mmHg	36 (33-40)	36 (32-39)	**0.014**	**0.013**
pO_2, mmHg	81 (67-97)	73 (61-90)	**<0.001**	**0.004**
pO_2/FiO_2	270 (206-362)	236 (126-333)	**<0.001**	**<0.001**
Clinical chemistry and haematology				
Haemoglobin, g/dl	13.1 (11.8-14.3)	13.6 (12.3-14.8)	**<0.001**	**<0.001**
White Blood Cells, $1x10^9$/L	7.41 (5.60-10.12)	6.41 (4.68-8.81)	**<0.001**	**<0.001**
Lymphocytes, $1x10^9$/L	1.01 (0.68-1.45)	0.87 (0.61-1.18)	**<0.001**	**<0.001**
Platelets, $1x10^9$/L	232 (181-312)	197 (155-246)	**<0.001**	**<0.001**
Creatinine, mg/dl	0.9 (0.7-1.1)	0.9 (0.7-1.2)	**0.033**	0.968
Sodium, mEq/L	138 (135-140)	137 (135-140)	0.096	0.142
Potassium, mEq/L	4.0 (3.7-4.4)	4.0 (3.7-4.3)	0.961	0.859
Total bilirubin, mg/dl	0.7 (0.5-0.9)	0.7 (0.5-0.9)	**0.004[b]**	**<0.001[b]**
Creatine-phosphokinase, IU/L	122 (63-219)	137 (74-324)	**0.002**	0.131
Lactate-dehydrogenase, IU/L	323 (250-433)	350 (269-472)	**0.001**	0.891
Aspartate aminotransferase, IU/L	40 (27-63)	46 (32-69)	**<0.001**	0.587
D-Dimer, ng/ml	1149 (653-2165)	975 (627-1707)	**0.013**	**0.036**
INR ratio	1.21 (1.13-1.34)	1.21 (1.13-1.32)	0.186	0.362
aPTT ratio	0.96 (0.89-1.04)	0.98 (0.90-1.07)	**0.006**	0.448
Fibrinogen, mg/dl	596 (480-754)	596 (490-730)	0.839	0.997
C-reactive protein, mg/L	89 (38-142)	102 (50-167)	**0.005**	**0.007**

[a]P adjusted for sex and age with linear regression.
[b]Bilirubin levels were higher in patients with negative RT-PCR test.
Data are shown as median and interquartile range. Crude comparisons were made with Mann-Whitney test, as appropriate. Data on RT-PCR results for SARS-CoV-2 detection were available in 1229 of the 1264 patients included in the study.

SUPPLEMENTARY TABLE 5

Comparison of the blood analysis on admission, after categorization of participants according to the presence of multimorbidity (≥2 chronic diseases).

	**Patients without multimorbidity (0-1 chronic diseases) (n=335)**	**Patients with multimorbidity (≥2 chronic diseases) (n=923)**	**P**	**P^a**	**P^b**	**β standardized for multimorbidity**
pH	7.45 (7.43-7.48)	7.44 (7.41-7.47)	**<0.001**	0.002	0.002	-0.100
Bicarbonate, mmol/L	25 (23-27)	25 (23-27)	0.182	0.662	0.682	-
pCO_2, mmHg	36 (33-39)	36 (33-40)	0.343	0.266	0.299	-
pO_2, mmHg	76 (64-90)	74 (62-93)	0.971	0.506	0.803	-
PaO_2/FiO_2, mmHg	286 (192-371)	243 (134-333)	**<0.001**	0.263	0.129	-
Haemoglobin, g/dl	14.0 (13.0-14.9)	13.2 (11.7-14.3)	**<0.001**	**<0.001**	**<0.001**	-0.152
White Blood Cell, $1x10^9$/L	6.60 (4.77-8.81)	6.78 (5.06-9.52)	0.093	0.430	0.570	-
Lymphocytes, $1x10^9$/L	0.94 (0.70-1.32)	0.88 (0.60-1.25)	**0.010**	0.558	0.679	**-**
Platelets, $1x10^9$/L	214 (177-271)	207 (159-266)	0.112	0.915	0.748	**-**
Creatinine, mg/dl	0.8 (0.7-1.0)	0.9 (0.7-1.3)	**<0.001**	**<0.001**	**<0.001**	0.111
Sodium, mEq/L	137 (135-139)	138 (135-140)	**0.008**	0.769	0.690	**-**
Potassium, mEq/L	4.0 (3.7-4.2)	4.0 (3.6-4.4)	**0.012**	**0.025**	**0.020**	0.074
Total bilirubin, mg/dl	0.7 (0.5-0.9)	0.7 (0.5-0.9)	0.483	0.074	0.061	-
Aspartate aminotransferase, IU/L	44 (33-69)	43 (29-68)	0.200	0.417	0.325	**-**
Creatine phosphokinase, IU/L	125 (73-265)	137 (68-296)	0.482	0.228	0.198	**-**
Lactate dehydrogenase, IU/L	350 (272-475)	337 (262-457)	0.452	0.749	0.592	**-**
D-dimer, ng/dl	801 (544-1498)	1096 (666-2008)	**<0.001**	0.159	0.144	**-**
Fibrinogen, ng/dl	612 (519-754)	596 (480-730)	**0.028**	0.114	0.154	**-**
INR ratio	1.21 (1.13-1.29)	1.21 (1.13-1.33)	0.147	0.090	0.109	**-**
aPTT ratio	0.96 (0.91-1.04)	0.97 (0.90-1.07)	0.187	0.341	0.382	-
C-reactive protein, mg/L	91 (44-154)	100 (49-163)	0.293	0.803	0.940	-

[a]P adjusted for age and sex or [b]age, sex and period of admission with linear regression, as appropriate.
Data are shown as median and interquartile range. Crude comparisons were made with Mann-Whitney test, as appropriate.
Data on multimorbidity were available for 1258 of the 1264 patients included in the study.

SUPPLEMENTARY TABLE 6

Comparison of the clinical course, treatments administered and outcomes, after categorization of participants according to the presence of multimorbidity (≥2 chronic diseases).

	**Patients without multimorbidity (0-1 chronic diseases) (n=335)**	**Patients with multimorbidity (≥2 chronic diseases) (n=923)**	**P**	**P[a]**	**P[b]**	**β standardized or Odds Ratio (95% Confidence Interval) for multimorbidity**
Clinical course						
Worst O_2 saturation during stay, %	92 (90-95)	91 (85-93)	**<0.001**	0.057	**0.015**	-0.075
Maximum O_2 flows during stay, %	35 (24-75)	44 (30-75)	**<0.001**	**0.028**	**0.011**	0.079
Worst arterial O_2 pressure, mmHg	62 (52-70)	59 (50-70)	**0.096**	0.688	0.401	-
Temperature peak during stay, °C	38.0 (37.2-38.8)	37.7 (36.9-38.5)	**0.183**	0.056	0.142	-
Non-invasive ventilation during stay	37 (11)	92 (10)	0.516	**0.037**	**0.026**	1.72 (1.07-2.76)
Therapies against COVID-19						
Antiviral drugs	181 (54)	424 (46)	**0.008**	0.548	0.645	**-**
Antibiotics	315 (94)	868 (94)	0.667	0.800	0.858	-
Azithromycin	275 (82)	683 (74)	**0.005**	**0.029**	0.090	**-**
Hydroxychloroquine	311 (63)	581 (63)	0.947	0.674	0.905	-
Steroid	33 (10)	203 (22)	**<0.001**	**<0.001**	**<0.001**	2.52 (1.62-3.90)
Enoxaparin	308 (92)	830 (90)	0.221	0.221	0.242	**-**
Fondaparinux	20 (6)	74 (8)	0.185	0.143	0.216	**-**
Outcome						
Intensive care unit	23 (7)	27 (3)	**0.004**	0.755	0.886	**-**
Death	40 (12)	277 (30)	**<0.001**	**0.036**	**0.015**	1.64 (1.10-2.45)

[a]P adjusted for age and sex or [b]age, sex and period of admission with linear or binary logistic regression, as appropriate.
Data are shown as median and interquartile range or percentages. Crude comparisons were made with Mann-Whitney test or chi-square test, as appropriate.
Data on multimorbidity were available for 1258 of the 1264 patients included in the study.

SUPPLEMENTARY TABLE 7

Comparison of the demographic, anamnestic and clinical features of patients, categorized according to the class of functional autonomy.

	**Patients with no autonomy/complete dependency in daily activities (n=210)**	**Patients with partial autonomy in daily activities (n=257)**	**Patients with complete autonomy in daily activities (n=784)**	**P[a]**	**β standardized or Odds Ratio (95% Confidence Interval) for autonomy**
Demography					
Age, years	86 (81-90)	82 (77-87)	66 (57-65)	**<0.001**	-
Female gender	134 (64)	113 (44)	306 (39)	**<0.001**	-
Weight, kg	62 (55-71)	72 (63-84)	80 (70-90)	0.063	
Anamnestic data					
Chronic diseases, n	4 (3-6)	4 (2-5)	2 (1-3)	**<0.001**	-0.248
CHA_2DS_2Vasc score	4 (3-5)	4 (3-5)	2 (1-3)	**<0.001**	-0.116
Systemic drugs, n	6 (4-8)	6 (3-8)	2 (0-5)	**<0.001**	-0.239
Hypertension	134 (64)	193 (75)	408 (52)	0.120	-
Diabetes	57 (27)	74 (29)	133 (17)	0.129	-
Obesity	10 (5)	28 (11)	110 (14)	0.305	-
Chronic heart disease	86 (41)	98 (38)	125 (16)	0.113	-
Dementia	132 (63)	41 (16)	8 (1)	**<0.001**	0.01 (0.01-0.03)
Clinical presentation of suspect COVID-19					
Symptom duration, days	4 (2-7)	5 (3-8)	7 (5-10)	**<0.001**	0.135
Cough	53 (25)	95 (37)	439 (56)	**<0.001**	2.48 (1.68-3.66)
Dyspnea	147 (70)	139 (54)	368 (47)	**0.020**	0.64 (0.44-0.93)
Fever	143 (68)	198 (77)	690 (88)	**0.031**	1.65 (1.05-2.60)
Other symptoms	42 (20)	62 (24)	110 (14)	**0.010**	0.55 (0.34-0.87)
O_2 saturation in room air on triage, %	90 (86-94)	92 (88-95)	94 (89-96)	0.392	-
Temperature on admission, °C	36.0 (36.0-36.8)	36.5 (36.0-37.4)	37.0 (36.0-37.8)	**<0.001**	0.155
O_2 flows administered on admission, %	36 (21-75)	36 (21-73)	30 (21-50)	0.664	-
CT visual score, %	30 (15-50)	30 (15-50)	30 (20-50)	**0.009**	0.101
Consolidations on chest CT	151 (72)	170 (66)	533 (68)	0.306	-
RT-PCR positive on admission	128 (61)	172 (67)	252 (67)	0.853	-

[a]p adjusted for age, sex and period of admission with linear or binary logistic regression, as appropriate.
Data are shown as median and interquartile range or numbers and percentages. Crude comparisons were made with Mann-Whitney or chi-square tests, as appropriate. Data on functional autonomy were available for 1251 of the 1264 patients included in the study.

SUPPLEMENTARY TABLE 8

Comparison of the blood analysis on admission, after categorization of participants according to functional autonomy.

	**Patients with no autonomy/complete dependency in daily activities (n=210)**	**Patients with partial autonomy in daily activities (n=257)**	**Patients with complete autonomy in daily activities (n=784)**	**P[a]**	**β standardized or Odds Ratio (95% Confidence Interval) for autonomy**
pH	7.43 (7.39-7.47)	7.44 (7.41-7.47)	7.45 (7.43-7.48)	**0.001**	0.122
Bicarbonate, mmol/L	24 (22-28)	24 (22-27)	25 (23-27)	0.281	-
pCO_2, mmHg	37 (33-42)	36 (32-29)	36 (33-39)	0.133	-
pO_2, mmHg	78 (64-104)	74 (62-94)	74 (62-91)	0.081	-
PaO_2/FiO_2, mmHg	238 (132-352)	237 (130-314)	258 (158-352)	0.181	-
Haemoglobin, g/dl	12.8 (11.0-14.2)	13.1 (11.7-14.3)	13.6 (12.4-14.7)	0.527	-
White Blood Cell, $1x10^9$/L	7.53 (5.55-10.44)	6.62 (4.97-9.54)	6.57 (4.93-8.99)	**0.024**	-0.080
Lymphocytes, $1x10^9$/L	0.90 (0.56-1.26)	0.82 (0.57-1.19)	0.93 (0.66-1.28)	0.522	-
Platelets, $1x10^9$/L	221 (164-280)	197 (152-261)	210 (169-269)	0.576	-
Creatinine, mg/dl	1.0 (0.8-1.6)	1.0 (0.8-1.3)	0.9 (0.7-1.1)	**<0.001**	-0.150
Sodium, mEq/L	139 (136-145)	138 (135-140)	137 (135-139)	**<0.001**	-0.247
Potassium, mEq/L	4.0 (3.6-4.5)	4.1 (3.7-4.5)	4.0 (3.7-7.3)	**0.028**	-0.078
Total bilirubin, mg/dl	0.6 (0.5-0.9)	0.7 (0.5-0.9)	0.7 (0.5-0.9)	0.733	-
Aspartate aminotransferase, IU/L	40 (26-67)	44 (29-72)	44 (32-67)	0.635	-
Creatine phosphokinase, IU/L	122 (51-356)	145 (72-340)	129 (72-261)	**<0.001**	-0.129
Lactate dehydrogenase, IU/L	314 (241-448)	336 (263-463)	346 (270-466)	0.980	-
D-dimer, ng/dl	1321 (826-2325)	1276 (784-3478)	860 (583-1449)	0.375	-
Fibrinogen, ng/dl	552 (450-666)	580 (460-708)	629 (502-754)	**<0.001**	0.149
INR ratio	1.21 (1.12-1.44)	1.22 (1.11-1.38)	1.21 (1.14-1.31)	**0.005**	-0.112
aPTT ratio	0.98 (0.88-1.10)	0.98 (0.89-1.07)	0.97 (0.90-1.04)	0.672	-
C-reactive protein, mg/L	89 (42-150)	105 (49-171)	98 (49-159)	**0.027**	0.077

[a]P adjusted for age and sex or bage, sex and period of admission with linear regression, as appropriate.
Data are shown as median and interquartile range. Crude comparisons were made with Mann-Whitney test, as appropriate.
Data on functional autonomy were available for 1251 of the 1264 patients included in the study.

SUPPLEMENTARY TABLE 9

Comparison of the clinical course, treatments administered and outcomes, after categorization of participants according to functional autonomy.

	**Patients with no autonomy/complete dependency in daily activities (n=210)**	**Patients with partial autonomy in daily activities (n=257)**	**Patients with complete autonomy in daily activities (n=784)**	**P[a]**	**β standardized or Odds Ratio (95% Confidence Interval) for autonomy**
Clinical course					
Worst O2 saturation during stay, %	91 (85-94)	91 (86-93)	92 (88-94)	0.291	-
Maximum O2 flows during stay, %	44 (30-75)	44 (32-75)	40 (28-75)	0.052	-
Worst arterial O2 pressure, mmHg	64 (55-76)	58 (48-69)	60 (49-69)	**0.005**	-0.118
Temperature peak during stay, °C	37.2 (36.0-38.0)	37.6 (36.8-38.3)	38.0 (37.2-38.8)	**<0.001**	0.132
Non-invasive ventilation during stay	0 (0)	13 (5)	110 (14)	**<0.001**	13.50 (4.34-41.92)
Therapies against COVID-19					
Antiviral drugs	53 (25)	113 (44)	439 (56)	**<0.001**	3.16 (2.15-4.65)
Antibiotics	193 (92)	242 (94)	737 (94)	0.879	-
Azithromycin	134 (64)	200 (78)	619 (79)	**0.030**	1.59 (1.05-2.43)
Hydroxychloroquine	107 (51)	167 (65)	510 (65)	**<0.001**	2.85 (1.91-4.24)
Steroids	42 (20)	54 (21)	141 (18)	0.100	-
Enoxaparin	185 (88)	231 (90)	713 (91)	0.109	-
Fondaparinux	19 (9)	20 (8)	55 (7)	.512	-
Outcome					
Intensive care unit	0 (0)	3 (1)	55 (7)	**0.007**	41.6 (2.8-615)
Death	90 (43)	87 (34)	133 (17)	**0.040**	0.64 (0.42-0.98)

[a]P adjusted for age, sex and period of admission with linear or binary logistic regression, as appropriate.
Data are shown as median and interquartile range or numbers and percentages. Crude comparisons were made with Mann-Whitney or chi-square tests, as appropriate. Data on functional autonomy were available for 1251 of the 1264 patients included in the study.

SUPPLEMENTARY FIGURE 1

Age distribution of patients admitted during the first (Feb 28-Mar 23, 2020) and the second phase (Mar 24-Jun 10, 2020) of the first pandemic wave in our institution.

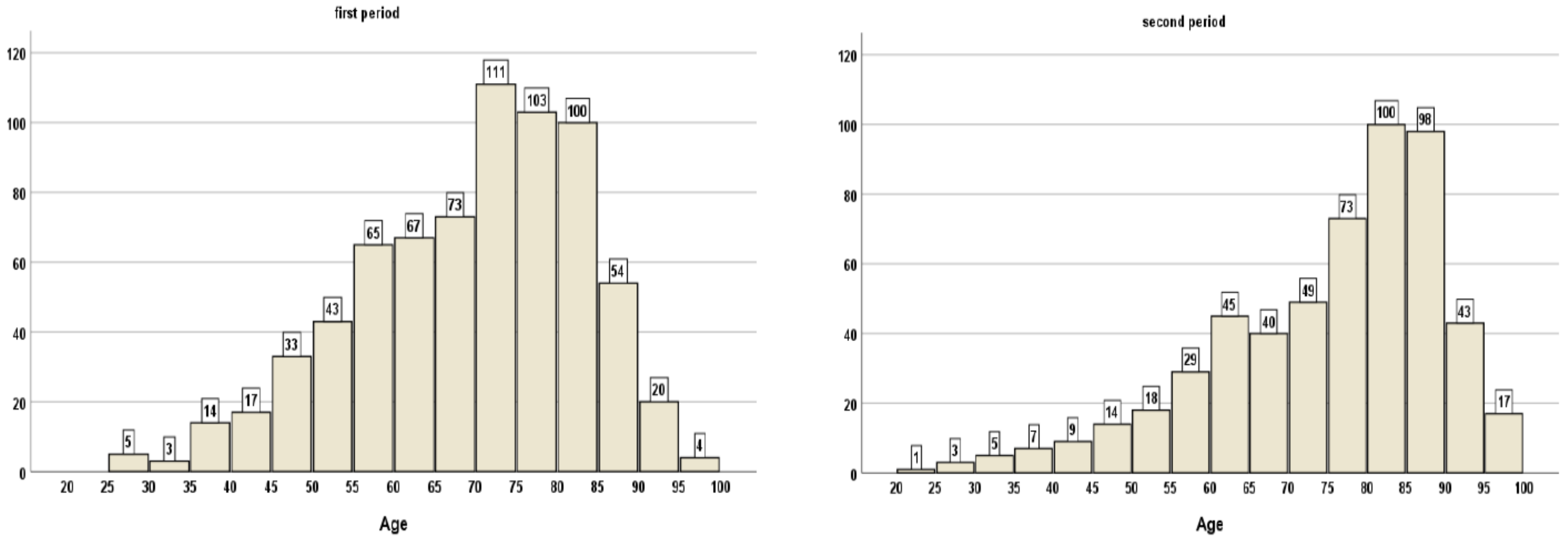

Supplement: Supplementary file 1 [file jcm-10-01115-s001.pdf]
